# STAT3, HIF-1, glucose addiction and Warburg effect

**DOI:** 10.18632/aging.100239

**Published:** 2010-12-03

**Authors:** James E. Darnell

**Affiliations:** The Rockefeller University, New York, NY 10065, USA

Oncogenes as the center of cancer research focused attention 30 years ago on signal transduction especially tyrosine phosphorylation. Discovery that the Jun oncoprotein was a site-specific DNA binding protein demanded a connection of signaling abnormalities with transcriptional control [1]. The cytokine responsive Jak-STAT pathway, discovered through studying α-interferon activation of STATs 1 and 2 by the Jak tyrosine kinases (Tyk2, Jak1, Jak2) was soon broadened to include the other five STATs [2]. The first description of STAT3 in 1994 [3] raised suspicion in the direction of cancer because IL-6 and EGF, already recognized to have cancer connections, were the originally recognized ligands that activated the STAT3 homodimer.

Less than a year later Richard Jove and colleagues made a convincing first link between STAT3 and oncogenesis [4]. Src-transformed cell lines all had persistently active STAT3 that was found to be required for Src-dependent oncogenic transformation of cultured cells [5,6]. Perhaps half or more of human tumors have long since been documented to contain persistently active STAT3 and human tumor cell lines depend on STAT3 for continued rapid growth and avoidance of apoptosis [7].

So while a STAT3 cancer connection has been extensively documented, what precisely does STAT3 do to promote or sustain cancer? Much work on STAT3 gene targets supports an anti-apoptotic role for STAT3 as well as a positive affect on cell growth.

Now, low and behold, STAT3 appears to be involved with the earliest recognized metabolic abnormality of cancer cells, energy derivation through glycolysis. Valeria Poli and a host of colleagues [8] have published a synthesis of previously established results plus many new experiments to provide convincing evidence that STAT3 transcriptional activity has an important role in establishing the addiction of tumor cells to glycolytic energy derivation and attendant glucose dependence - the Warburg effect [9, 10].

Most of the new experiments [8] use mice in which the wild type STAT3 gene is replaced by a mutation originally produced in our laboratory. We designed a STAT3 molecule we thought might provide constitutive activity by inserting cysteines in a relatively unstructured region of the SH2 domain [11]. Believing we had such a mutation because of high transcriptional signals without cytokine stimulation by the transfected mutant compared to wild type, Jackie Bromberg went on to show this mutant protein, named STAT3C, was capable of transforming cultured, partially transformed fibroblasts (NIH3T3 and rat 3Y1 cells). These transformed cells grew into tumors in nude mice. Many laboratories around the world have used the STAT3C construct that consistently augments transformation as we had found.

However, we never obtained (attempts were made) to get a Y705F mutant of STAT3C and did not demonstrate therefore that the mutant required phosphorylation of Y705 just as does the wild type in order to function as a oncogene. Peter Shaw however has corrected our error. STAT3C indeed requires Y705 phosphorylation to be active [12]. Shaw and Li showed that the STAT3C phosphodimer binds DNA better than the wild type, a characteristic that allows it to remain phosphorylated in the nucleus for a longer period of time after activation. Thus STAT3C is simply what the bacterial geneticists were calling an “up” mutation several decades ago. There is still no dispute that STAT3C can act as a cooperating oncogene. And Demaria et al. [8] show definitive effects in MEFs (mouse embryo fibroblasts) of STAT3C/C compared to STAT3 WT including presence in the cell nucleus without a known cytokine activation, longer nuclear retention after IL-6 treatment, faster growth, and resistance to apoptosis induced by four different agents. The activity of STAT3C is presumably due to the low level of STAT3 activation of cells growing in normal serum containing medium, a situation proven earlier by blocking phosphatase activity in the absence of a cytokine ligand and observing STAT1 and STAT3 DNA binding [13,14].

The Warburg type tumor metabolism in STAT3C/C cells depends on an increase in levels of HIF1 α that is at least partly due to increased STAT3 dependent transcription shown earlier by Niu et al. [15] with further new evidence in STAT3^C/C^ MEFs. And HIF1α increases transcription of all known glycolytic enzymes. STAT3c/c activity also increases mRNA for a set of proteins, including PDK-l (pyruvate dehydrogenase kinase 1) which blocks the shuttling of pyruvate into the citric acid cycle in mitochondria sending it into the glycolytic pathway with resulting increased lactic acid production. With the increased dependence on glycolysis STAT3^C/C^ cells require high glucose to avoid apoptosis. A decreased mitochondrial oxidative phosphorylation attendant to presence of STAT3^C/C^ likely depends on a lowered amount of mRNA for mitochondrial proteins, e.g. ATP synthase, for the G subunit of the mitochondrial FO complex, for fumarate hydratase.

The authors discuss the recent findings of Andy Larner and David Levy [16,17] that establish the presence of non-tyrosine phosphorylated STAT3 in mitochondria that plays a required role in transformation by the Ras oncogene. It is the phosphorylation of serine 727, which in the nucleus is required for maximal transcriptional stimulation, that is important in both the Ras transformation of cells as well as in the maximal functioning of mitochondrial oxidative phosphorylation.

A major point made in the Demaria et al. paper is that the metabolic effect brought about by phosphorylating tyrosine 705 activates STAT3 in the cell nucleus (as must occur at a low level in STAT3^C/C^) is a separate set of events from the serine 727 phosphorylation inside the mitochondria. Thus there is no contradiction between the two sets of findings but emphasizes STAT3 has multiple roles in cell transformation. The powerful message of the newest results is that STAT3 is involved in the time-honored characteristic of cancer cells, dependence on glycolytic energy derivation.
